# Transient cortical weakness following cerebral angiography: A new syndrome

**DOI:** 10.1016/j.radcr.2024.02.080

**Published:** 2024-03-21

**Authors:** Akash P. Kansagra, Richard Pham, Arindam R. Chatterjee, Christopher J. Moran

**Affiliations:** a Mallinckrodt Institute of Radiology, Washington University School of Medicine, St. Louis, MO; b Department of Neurological Surgery, Washington University School of Medicine, St. Louis, MO; c Department of Neurology, Washington University School of Medicine, St. Louis, MO; d University of California Riverside School of Medicine, Riverside, California

**Keywords:** Brain, Angiography, Contrast, Neurotoxicity syndromes

## Abstract

Transient cortical blindness is a known complication of iodinated contrast administration and is believed to reflect osmotic injury or autoregulatory dysfunction of the posterior circulation. Here, we report 2 cases of postangiography transient cortical *weakness*, a rare clinical analog to transient cortical blindness that affects the anterior circulation. The symptoms, timeline, and imaging findings of transient cortical weakness are distinct from more common post-procedural complications such as acute ischemic stroke or transient ischemic attack.

## Introduction

Transient cortical blindness is a well-described complication of intravascular administration of iodinated contrast [1], [2], [3], [4]. This syndrome preferentially affects the posterior circulation territories of the brain, particularly the visual cortex, resulting in complete binocular vision loss shortly after contrast administration [1], [2], [3], [4]. Here, we report two cases of a much rarer contrast-induced syndrome, transient cortical *weakness*, which affects the anterior circulation and mimics a middle cerebral artery stroke syndrome [5,6]. In each case, the symptoms, timeline, and imaging were inconsistent with acute ischemic stroke or transient ischemic attack. All patients spontaneously regained neurological function over days.

## Case reports

### Case 1

A 39-year-old female presented for treatment of an incidentally discovered left superior hypophyseal aneurysm. She underwent uneventful stent-assisted coil embolization of this aneurysm using 154 cc of Optiray 320 (Liebel-Flarsheim Company LLC, Raleigh, NC, USA). One hour after the procedure, the patient reported sudden onset numbness and tingling of the right hand, arm, and face, which rapidly progressed to right arm weakness, aphasia, and altered mental status.

**Fig. 1:**
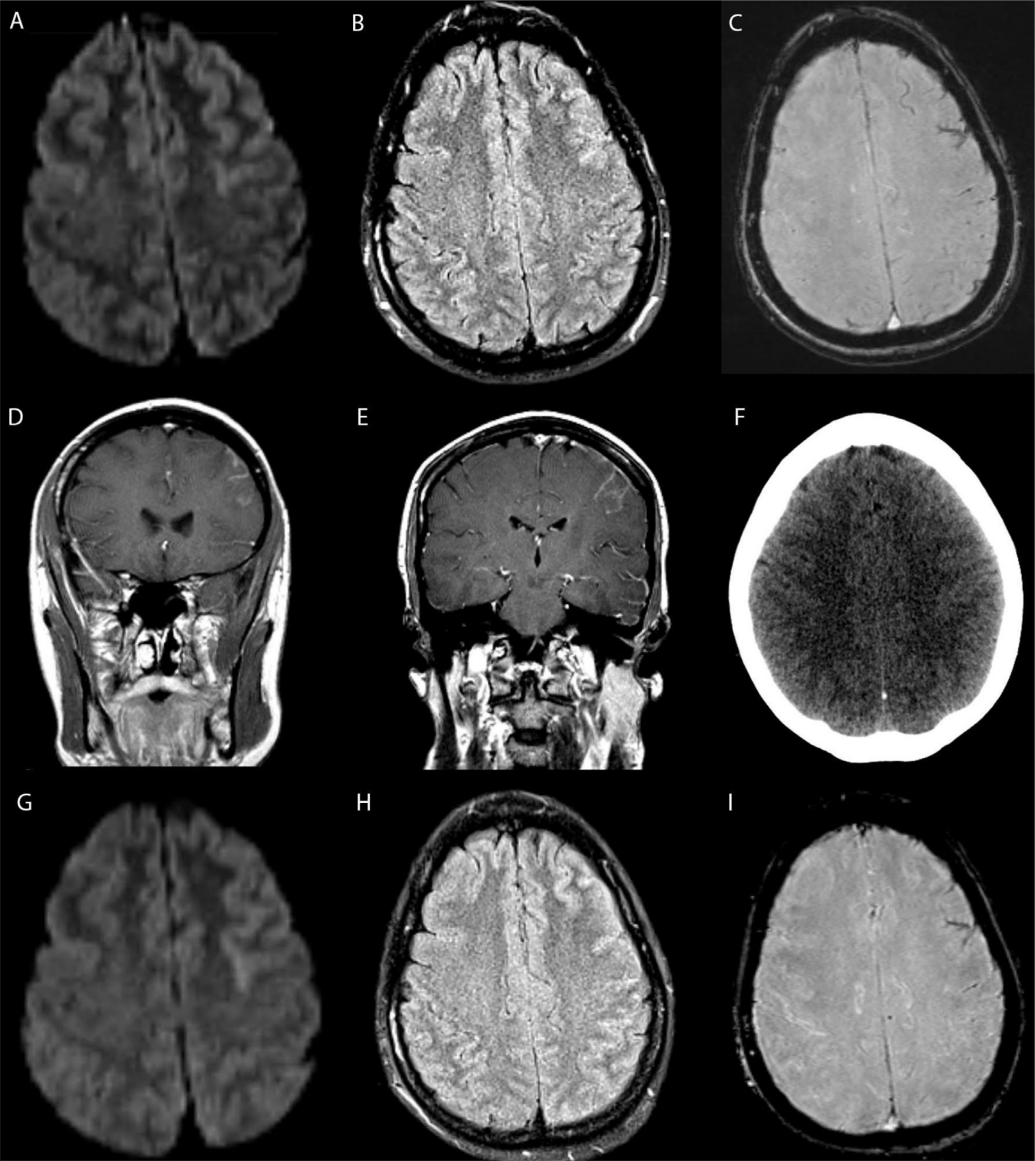
Imaging studies from Case 1. 3.0 Tesla MRI performed 2 hours after symptom onset demonstrates no acute infarction on DWI (A) and subtle FLAIR non-suppression (B), susceptibility artifact (C), and contrast enhancement (D, E) in the left cerebral hemispheric sulci. Non-contrast CT (F) performed 13 hours after symptom onset demonstrates subtle edema and sulcal effacement in the left cerebral hemisphere. 1.5 Tesla MRI performed 14 hours after symptom onset again demonstrates no acute infarction on DWI (G) but FLAIR non-suppression (H) and susceptibility artifact (I) in a left fontal sulcus, compatible with extravasated contrast or trace subarachnoid blood.

Repeat cerebral angiography performed 20 minutes after symptom onset demonstrated no abnormalities. MRI performed 2 hours after symptom onset demonstrated no acute infarction but did demonstrate subtle FLAIR nonsuppression, susceptibility artifact, and contrast enhancement within the sulci of the left cerebral hemisphere (Fig. 1). Noncontrast CT performed 13 hours after symptom onset showed subtle edema of the left frontal cortex with asymmetric effacement of the sulci. Repeat MRI performed 14 hours after symptom onset demonstrated no acute infarction but did again demonstrate FLAIR hyperintensity and susceptibility artifact in a left frontal sulcus thought to represent extravasated contrast or subarachnoid blood.

An idiosyncratic contrast reaction was suspected based on the continued absence of imaging findings of stroke or vessel occlusion, and the patient was managed expectantly. Neurological deficits spontaneously improved on postoperative day 1 and nearly resolved by postoperative day 2, with mild right shoulder weakness and numbness of the left posterior thigh that were resolved at the time of next follow up.

### Case 2

A 76-year-old female presented for follow up angiography of a cavernous left internal carotid artery aneurysm treated with coil embolization 6 months prior. Cerebral angiography was performed using 90 mL of Optiray 320. Two hours later, the patient developed sudden onset right hemiparesis and aphasia.

**Fig. 2:**
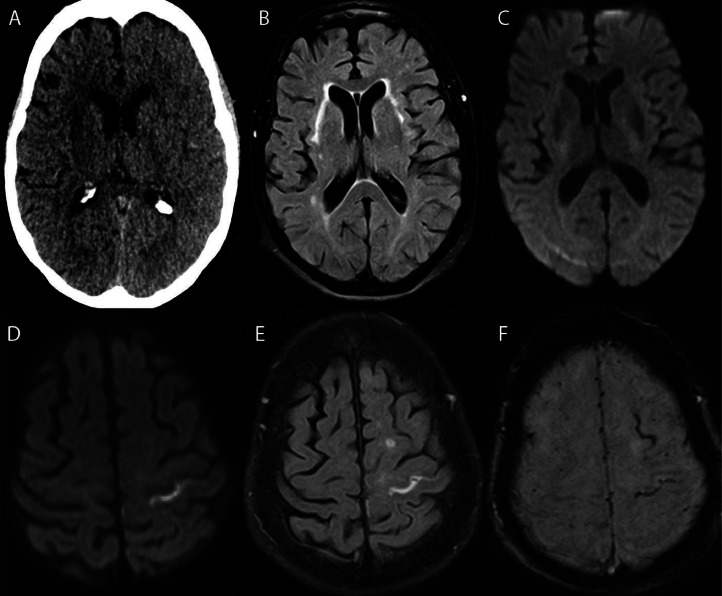
Imaging studies from Case 2. Non-contrast CT (A) performed 30 minutes after symptom onset demonstrates profound left hemispheric edema. 3.0 Tesla MRI performed 48 hours later demonstrates resolved edema on FLAIR images (B) and no acute infarction on DWI (C) in the previously affected regions; in the left central sulcus, DWI (D), FLAIR (E), and susceptibility-weighted imaging (F) demonstrate diffusion restriction, linear FLAIR non-suppression, and susceptibility artifact compatible with extravasated contrast or trace subarachnoid blood.

Noncontrast CT and CTA was performed 30 minutes after symptom onset. This demonstrated edema in the left hemispheric edema without vessel occlusion (Fig. 2). MRI performed 2 days after symptom onset revealed no acute infarction but did demonstrate FLAIR hyperintensity and susceptibility artifact within the left central sulcus, thought to represent extravasated contrast or subarachnoid blood.

The patient was managed expectantly. Neurological deficits spontaneously improved on post-procedure day 2 and nearly completely resolved by day 5. However, the patient had persistent mild cognitive dysfunction.

## Discussion

We describe 2 cases of a rare clinical syndrome of transient cortical weakness. This syndrome appears to have an analogous time course, imaging, and outcome as the more familiar contrast-induced syndrome of transient cortical blindness. However, it involves the middle cerebral artery territory rather than the posterior cerebral artery territory, with corresponding differences in neurological symptoms.

Transient cortical weakness must be distinguished from more common and serious neurological complications such as middle cerebral artery stroke, transient ischemic attack, or intracranial hemorrhage. Imaging can be an early discriminator between these conditions. Positive imaging findings of transient cortical weakness include profound edema very early in the course of the syndrome along with FLAIR hyperintensity and/or susceptibility artifact in the sulci of the affected territory. These findings are not typically seen in ischemic stroke [3,4]. Negative imaging findings, such as absence of diffusion restriction and parenchymal susceptibility artifact on MRI, can exclude acute ischemic stroke or parenchymal hemorrhage [3,4].

The pattern of imaging seen in transient cortical weakness bears similarities to posterior reversible encephalopathy syndrome, which has been implicated as a potential explanation for transient cortical blindness [3]. The imaging findings in posterior reversible encephalopathy syndrome, including parenchymal edema and leptomeningeal enhancement, are thought to reflect altered cerebrovascular autoregulation and/or endothelial dysfunction resulting in disruption of the blood-brain barrier [1,3,7]. Due to the similarity of imaging findings in transient cortical weakness and transient cortical blindness, we speculate that both are manifestations of a similar physiological derangement, albeit in different territories. Despite the greater susceptibility of the posterior circulation to this derangement, which is speculated to result from reduced sympathetic innervation [1], our 2 cases are an important reminder that similar phenomena may occur in other vascular territories and produce a constellation of symptoms that may be challenging to recognize as an idiosyncratic contrast reaction.

The risks factors for transient cortical weakness in our patients are unknown, as both had previously undergone uneventful neurointerventional procedures. Treatment is also unknown but should ostensibly involve avoidance of further iodinated contrast administration before symptom resolution. In our patients, symptoms and imaging findings of transient cortical weakness improved spontaneously and nearly completely over several days. Early recognition of the clinical and imaging features of transient cortical weakness may help to avoid unnecessary contrast administration or invasive procedures to work up more common post-treatment complications.

## Conclusion

We report 2 cases of postangiography transient cortical weakness, an anterior circulation analog of the more widely known transient cortical blindness. Both cases had symptoms, timeline, and imaging findings that were inconsistent with ischemic stroke or transient ischemic attack. Transient cortical weakness should be considered in patients with stroke-like symptoms referable to the anterior circulation after contrast administration. Both patients had dramatic and spontaneous neurological improvement within days.

## Patient consent

Written and informed consent was obtained for publication from the patients.
